# Analysis of Necroptosis-Related Genes and Immune Cell Infiltration in Infantile Hemangioma

**DOI:** 10.2174/0118715303388166250901114424

**Published:** 2025-09-16

**Authors:** Yunpeng Xu, Jiajie Chen, Zhihao Huang, Rui Zhao, Can Jin, Zichen Li, Shengxiu Liu

**Affiliations:** 1 Department of Dermatovenerology, The First Affiliated Hospital of Anhui Medical University, Hefei, Anhui, China

**Keywords:** Infantile hemangioma, necroptosis-related genes (NRGs), immune cell filtration, bioinformatics analysis, diagnostic model, interaction network

## Abstract

**Introduction:**

Infantile hemangioma (IH) is the most prevalent benign tumor in neonates, yet the role of necroptosis in its pathogenesis remains underexplored. This study aimed to investigate necroptosis-related gene expression patterns in IH, identify critical biomarkers, and develop a diagnostic model to enhance precision medicine approaches.

**Methods:**

Gene expression data from GSE127487 and GSE100682 datasets were analyzed to screen necroptosis-related differentially expressed genes (NRDEGs). A diagnostic model was constructed using a support vector machine (SVM) and least absolute shrinkage and selection operator (LASSO) regression. Model validity was assessed *via* receiver operating characteristic (ROC) curve analysis. mRNA interaction networks and immune cell associations were explored using MCPCounter algorithms and single-sample gene set enrichment analysis.

**Results:**

Seventy-four NRDEGs were identified, with six key genes highlighted. Network analysis revealed six miRNAs and 47 transcription factors associated with these genes. Additionally, six key genes showed associations with eight distinct immune cell types, suggesting potential roles in regulating the immune microenvironment.

**Discussion:**

In this study, bioinformatics was employed to analyze data, construct diagnostic models, and identify key genes.

**Conclusion:**

The six key genes may serve as reliable biomarkers for IH, offering insights into precise diagnosis and personalized therapeutic strategies. This study advances understanding of necroptosis mechanisms in IH and their interplay with immune cells.

## 1 INTRODUCTION

Infantile hemangioma (IH) is a common benign vascular neoplasm that affects 5-10% of mature newborns [1]. The incidence of hemangiomas in low-birthweight preterm infants can increase up to 23% [2]. Although IH is a benign tumor, it can lead to serious complications because almost 60% of IH cases occur in the neck, face, and head regions. Hemangioma occurring around the eyes can cause refractory astigmatism, nearsightedness, strabismus, and other complications [3], while IH occurring on the tongue and nasopharynx can obstruct the airway, which can lead to death by suffocation in severe cases [4]. IH not only seriously affects aesthetics but also causes tissue and organ deformities and dysfunction, which reduces patients' quality of life and can even lead to psychological disorders. Conventional treatments include cryosurgery, surgery, and local drug injections, all of which have varying degrees of limitation. These methods often result in serious adverse effects, such as post-treatment scarring, ulcers, and pigmentation abnormalities. Currently, the first-line drugs used for the treatment of IH are propranolol and glucocorticoids; however, concerns regarding their safety and resistance persist. Propranolol, a nonselective beta-adrenergic receptor blocker, can cause serious adverse effects, such as hypotension, central nervous system toxicity, convulsions, bronchospasm, and hypoglycemia. Glucocorticoids, in contrast, are prone to adverse effects, such as personality changes, fungal infections, hypertension, Cushing's syndrome, and gastrointestinal discomfort. The unsatisfactory effects of laser and systemic medication and the residual damage caused by cryosurgery, surgery, and local drug injections can lead to psychological isolation and introversion, thus affecting the patient's physical and mental health and causing tremendous psychological pressure on the patient and their family. Therefore, more in-depth and comprehensive research is required to explore novel diagnostic and treatment options to facilitate advancements in the accurate diagnosis and personalized treatment of this illness.

Necroptosis, which is regulated independently by aspartate, is a distinct form of programmed cell death. Cell necrosis exhibits typical apoptotic features, including cellular swelling and membrane rupture, which release considerable amounts of damage-associated molecules, inflammatory cytokines, and chemokines [5]. Necroptosis is involved in various pathophysiological mechanisms, including infections, liver diseases, and neurodegenerative conditions [6]; therefore, controlling it is vital for maintaining immune function [7]. Emerging research has underscored the involvement of necroptosis in tumor pathogenesis and metastasis [8]. It can potentially hinder the growth, advancement, and spread of tumors. Furthermore, agents that induce necroptosis with immune checkpoint inhibitors can synergistically suppress tumors [9]. The development of IH relies on angiogenesis. Necroptosis-induced cell death may impair the function of key endothelial cells, thereby reducing the functionality of the tumor's blood supply and hindering its growth. During the natural regression of IH, inflammation facilitates the clearance of abnormal endothelial cells and fibrotic tissue. Necroptosis releases damage-associated molecular patterns (DAMPs), attracting macrophages and other immune cells to support tumor involution. Necroptosis plays a dual role in IH, regulating tumour growth and inflammation to limit progression and providing molecular and inflammatory mediators as potential therapeutic targets. Further investigation into the specific mechanisms of necroptosis in the development and regression of hemangiomas may pave the way for novel treatment strategies.

To investigate the involvement of necroptosis in IH development, we obtained a dataset on IH from the Gene Expression Omnibus (GEO) database and a set of necroptosis-related genes (NRGs) from GeneCards. Based on this, support vector machine (SVM) analysis was combined with the least absolute shrinkage and selection operator (LASSO) diagnostic model to collaboratively identify key genes. Additionally, in-depth analyses of functional and pathway enrichment revealed the biological mechanisms and pathways in which these NRGs might be involved in IH pathogenesis. By leveraging a single-sample gene set enrichment analysis (ssGSEA), immune infiltration analysis was carried out, and networks illustrating mRNA-TF and mRNA-miRNA interactions were generated. The identification of key genes not only revealed the potential role of necroptosis in IH but also provided potential targets for new molecular markers and therapeutic interventions for clinical diagnosis.

## 2 MATERIALS AND METHODS

### 2.1 Data

The IH datasets, GSE127487 [10] and GSE100682 [11], were retrieved from the GEO database [12] (https://www.ncbi.nlm.nih.gov/geo/) utilizing the R package GEOquery [13]. Among them, 18 IH (group: IH) and 5 normal (group: Normal) microarray gene expression profiles were included in the GSE127487 dataset. Normal control tissue was obtained from healthy skin containing normal vascular structures at the same site of origin as the hemangioma sample and served as a physiological control for the hemangioma tissue. The GSE127487 dataset exclusively contained samples derived from *Homo sapiens*. The chip and the platform used for the dataset were a GPL10558 Illumina HumanHT-12 V4.0 Expression BeadChip.

The GSE100682 dataset contained the miRNA expression profile data of three IH samples (group: IH) and three normal samples (group: Normal). The GSE100682 dataset exclusively contained samples from *Homo sapiens*. The chip platform used for this dataset was the GPL21572 (miRNA-4) Affymetrix Multispecies miRNA-4 Array (ProbeSet ID version). Specific information on the samples is provided in Table **S1**.

GeneCards [14] (http://www.genecards.org/), a database with an extensive collection of data related to human genes, was used for the acquisition of NRGs. “Necroptosis” was used as the keyword for our search, yielding a total of 647 NRGs. The detailed information is provided in Table **S2**.

### 2.2 Necroptosis-related Differentially Expressed Genes (NRDEGs) Linked to IH

Using the limma program in R, we investigated the variations in gene expression between the IH and normal groups. The criteria for identifying differentially expressed genes (DEGs) were established at |logFC| > 0 and adjusted to *p* < 0.05. DEGs with logFC > 0 and adj. *p* < 0.05 were classified as upregulated, while those with logFC < 0 and adj. *p* < 0.05 were categorized as downregulated.

NRDEGs related to IH were identified through differential analysis of the GSE127487 dataset, with all |logFC| > 0 and adj. *p* < 0.05. Then, the intersection of DEGs and NRGs was obtained, and Venn diagrams were used to identify the NRDEGs. Furthermore, the differential analysis results were used to create volcano plots and identify differences using the R package ggplot2.

### 2.3 Functional Enrichment Analysis (GO) and Pathway Enrichment (KEGG) Analysis

Gene Ontology (GO) analysis [15] is widely used for conducting comprehensive functional enrichment investigations encompassing biological processes (BP), cell components (CC), and molecular functions (MF). The Kyoto Encyclopedia of Genes and Genomes (KEGG) [16] is a well-known database that compiles data on drugs, diseases, biological pathways, and genomes. In this study, the NRDEGs were analyzed using GO and KEGG pathway enrichment analyses implemented utilizing the R package clusterProfiler [17]. The inclusion criteria for significance were established at adj. *p* < 0.05 and FDR value (q-value) < 0.05. These values were deemed statistically significant by applying the Benjamin-Hochberg (BH) method for the adjustment of the *p*-value.

### 2.4 Gene Set enrichment Analysis (GSEA) and Gene Set Variation Analysis (GSVA)

GSEA [18] is a technique utilized to ascertain the patterns in which genes are distributed within a pre-specified gene set. In this study, they were organized in a gene table that was arranged based on phenotypic correlation, thereby determining their impact on the phenotype contribution. We initially arranged genes in the GSE127487 dataset according to their logFC values. All the genes in the dataset were subjected to GSEA utilizing the R clusterProfiler package. Additionally, the criteria set included a seed set in 2020, 1000 iterations, as well as a minimum and maximum of 10 and 500 genes, respectively, within each set. Gene collection c2.cp.all.v2022.1. Hs.symbols.gmt (All Canonical Pathways) from the Molecular Signatures Database (MSigDB) [19] was used for the GSEA. The screening criteria for the GSEA comprised an adj. *p* < 0.05 and FDR (q) < 0.25, with the BH methods used for the adjustment of the *p*-value.

GSVA [20] is a non-parametric, unsupervised analytical method that serves as a conversion tool for gene expression matrices among various samples, transforming them into gene set expressions across the samples. An IH quantitative matrix was used to evaluate gene set enrichment in the cellular transcriptome and determine the presence of enriched pathways across distinct samples. The reference gene set 'h.all.v7.4. symbols.gmt' was sourced from the MSigDB database (https://www.gsea-msigdb.org/). To uncover the functional variances between groups within the GSE127487 dataset, genes were scrutinized for significance based on a *p* < 0.05, with the BH method employed for *p*-value corrections. The expression matrix was subjected to GSVA, and the enriched pathways were calculated based on the GSVA results.

### 2.5 Development of an IH Diagnostic Model

The NRDEGs were subjected to logistic regression analysis to establish the IH diagnostic model using the GSE127487-derived data. When the dependent variable was binary (IH and normal groups), the association between the independent variables and the dependent variables was determined *via* logistic regression. Using logistic regression modeling, the NRDEGs were scrutinized based on a significance threshold of *p* <0.05. The involvement of necroptosis in the logistic regression model was graphically illustrated in a forest plot [21]. Additionally, the expression patterns of the NRDEGs were categorized for further analysis.

The NRDEGs included in the logistic regression were subjected to the LASSO analysis utilizing the R package glmnet [22]. Parameters, such as set.seed (500) and family=”binomial”, were specified during the analysis. LASSO regression analysis is based on linear regression analysis, by adding a penalty term (lambda × absolute value of slope), which reduces the overfitting of the model and improves the generalization ability of the model. The results of LASSO regression analysis were visually displayed through diagnostic model diagrams and variable trajectory diagrams. The results of the LASSO regression analysis constituted the IH diagnostic models, and the NRDEGs included in these models are referred to as Model Genes. Finally, the LASSO risk score was calculated based on the risk coefficient of the LASSO regression analysis. The risk score calculation formula (1) is as follows [23]:


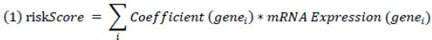


Following this, using the SVM [24] algorithm, we utilized NRDEGs identified from the logistic regression model to generate an SVM model. Then, the SVM model was refined by selecting NRDEGs based on the optimal number of genes that yielded the highest accuracy and minimized the error rate.

For subsequent analysis, we chose genes obtained from the intersection of the NRDEGs screened by the LASSO regression and SVM analyses.

### 2.6 Validation of the Diagnostic Model for IH

Subsequently, we formulated a nomogram specifically for NRDEGs utilizing the rms R package. A nomogram graphically displays the intricate relationships between several independent variables within a rectangular coordinate system. It was developed through a multifactor regression analysis, where a specific scale was assigned to each score and the variables within the model were characterized. The cumulative total score derived from the nomogram serves as a reliable indicator of the likelihood of a particular event.

For the assessment of the clinical prediction models, molecular markers, and diagnostic evaluations, we conducted Decision Curve Analysis (DCA). A DCA plot for evaluating the accuracy and reliability of the logistic regression models was developed utilizing the R package ggDCA.

Additionally, the R package pROC was applied to create a receiver operating characteristic (ROC) curve for the GSE127487 dataset, and the area under the curve (AUC) was computed to predict IH incidence. The AUC of the ROC curve typically falls within the range of 0.5 to 1, with a higher AUC indicating greater diagnostic power.

### 2.7 Expression Difference Analysis of the Key Genes

To uncover the possible underlying processes of various genes in group IH, along with the associated biological traits and pathways, we conducted the Mann–Whitney U test (also known as the Wilcoxon rank-sum test) to identify IH samples in the GSE127487 dataset, focusing on key genes. The variance in expression between the IH group (grouping: IH) and the normal sample group (grouping: Normal) was examined, followed by the presentation of the differential analysis outcomes utilizing the R ggplot2 package to develop a comparative chart.

Subsequently, we examined key genes based on the outcomes of the expression difference analysis, delved deeper into the ROC curves of these key genes within the GSE127487 dataset, and presented findings. The ROC curve [25] was used for identifying the most appropriate model, eliminating less optimal models, and determining the ideal threshold value for the same model. Notably, the ROC curve is a comprehensive platform that reflects the ongoing variables of specificity and sensitivity. Additionally, the method used in this assay reflected the interaction between specificity and sensitivity. Typically, the range of AUC of the ROC curve is 0.5–1, with diagnostic power increasing as the value approaches 1. Accuracy decreases when the AUC value is between 0.5 and 0.7, increases between 0.7 and 0.9, and improves when the value exceeds 0.9. Notably, the ROC curve for the selected key genes within the GSE127487 dataset was plotted utilizing the pROC package in R, after which the AUC was computed to ascertain the diagnostic impact of NRDEG expression on the prognosis of IH patients.

### 2.8 Interaction Network for mRNA-miRNA and mRNA-TF

The ENCORI database [26] (https://starbase.sysu.edu.cn/), the third version of the StarBase database, contains a wide range of interaction datasets, including RBP-ncRNA, ncRNA-RNA, miRNA-mRNA, RNA-RNA, miRNA-ncRNA, and RBP interactions. These datasets were curated through rigorous data mining of CLIP-seq and degradome-seq data (specifically for plants), presenting a range of visual interfaces for evaluating miRNA targets. Using the ENCORI database, we predicted the interactions between miRNAs and key genes (mRNA) and intersected these identified miRNAs with DEGs (miRNAs) from GSE100682 to ascertain the intersection of miRNAs. Cytoscape is an open-source software platform for visualizing complex networks and integrating them with attribute data. It is widely used in the biomedical field, particularly for analyzing molecular interaction networks and biological pathway analysis [27].

Subsequently, we depicted the network of interactions between mRNA and miRNAs by Cytoscape, shedding light on their interplay in regulatory mechanisms.

The CHIPBase database [28] (version 3.0; https://rna.sysu.edu.cn/chipbase/) contains DNA-binding protein chromatin immunoprecipitation (ChIP)-seq data, revealing the binding motif matrices as well as the corresponding sites and enabling the prediction of transcriptional regulatory interactions between genes and transcription factors (TFs). Using the CHIPBase version 3.0, we conducted a targeted search to identify TFs interacting with key mRNA genes.

### 2.9 Analysis of Immune Infiltration (Single-Sample Gene Set Enrichment Analysis [ssGSEA])

ssGSEA [29] allows for the quantification of the relative abundance of each immune cell infiltrate. First, any invading immune cell, including regulatory T cells, activated dendritic cells (DCs), gamma-delta T cells, natural killer (NK) cells, activated CD8 T cells, and several other types of immune cells in humans, was marked. The ssGSEA enrichment score serves as an indicator of the relative prevalence of immune cell infiltrates per sample, culminating in the compilation of an immune cell infiltration matrix. Samples with *p* < 0.05 were retained for further assessment, while others were omitted. Comparative analyses were performed to highlight disparities in the infiltration levels of immune cells between the IH and normal subgroups, as depicted in the group comparison diagrams. Heatmaps representing the association of immune cell subsets with necroptosis in the IH group were created utilizing the pheatmap package in R. The correlation analysis outcomes between the NRDEGs and immune cells were also presented.

### 2.10 Immune Infiltration Analysis (MCPCounter)

The MCPCounter [30] was adopted to measure the infiltration levels of immune cells and other stromal cells into the tissue from the expression data. In particular, the MCPCounter provides quantification data for eight distinct immune cell groups, which include CD4+ and CD8+ T cells, NK cells, B lymphocytes, monocytes, DCs, neutrophils, and toxic lymphocytes, which include cytotoxic innate lymphoid NK cells and CD8+ T cells. This method was also used to calculate the levels of two non-immune stromal cells: fibroblasts and endothelial cells. The Spearman correlation analysis used estimates of MCPCounter abundance and antigen gene activity, with statistical significance attributed to *p* < 0.05.

### 2.11 Statistical Analysis

All operations involving the processing and analysis of data were completed using R software (v 4.2.2). The comparative analysis of two sets of normally distributed continuous variables was conducted utilizing the independent Student’s *t*-test unless specified otherwise. For variables not conforming to a normal distribution, we applied the Mann–Whitney U test (also known as the Wilcoxon rank-sum test) for significance. For comparative analysis involving more than two groups, we carried out the Kruskal–Wallis test. Spearman’s correlation analysis was executed to obtain the correlation coefficients between the various molecules. All reported *p*- values were bidirectional, with values below 0.05 considered statistically significant.

## 3 RESULTS

### 3.1 Technology Roadmap

The flow diagram of the study is displayed in Fig. (**1**).

### 3.2 Dataset Correction

Primarily, this study employed bioinformatics techniques to study the biological traits of IH. A comprehensive analysis workflow is illustrated in Fig. (**2**). The GSE127487 (Figs. **2a**–**b**) was standardized, and annotated probes and other data cleaning processes were conducted. Thereafter, a box plot of the data distribution was generated, pre- and post-normalization. After standardization, the expression patterns across various samples in the dataset generally remained uniform [31].

### 3.3 NRDEGs Associated with IH

As a result, 2,098 genes in the GSE127487 dataset were identified as DEGs with a logFC| > 0 and adj. *p* < 0.05 threshold; of which, 1,569 genes were upregulated in expression (logFC > 0 and adj. *p* < 0.05), while the remaining genes exhibited downregulated expression (logFC < 0 and adj. *p* < 0.05). Subsequently, a volcano plot (Fig. **3a**) was created using the differential analysis outcomes of this dataset.

Variations in gene expression between the IH and normal groups within the GSE100682 dataset were assessed utilizing the R limma package to detect the genes that display distinct expression patterns in these two groups [32]. Following the analysis, 38 genes in the GSE100682 dataset satisfied the criteria for DEGs (logFC| > 0 and adj. *p* < 0.05). Notably, all these genes exhibited upregulated expression (logFC > 0 and adj. *p* < 0.05), as presented in Table **S3**.

To obtain the NRDEGs, the intersection profiles of all the obtained DEGs with a |logFC| > 0 and adj. *p* < 0.05 and NRGs were drawn, after which a Venn diagram (Fig. **3b**) was plotted. A total of 74 NRDEGs were identified (Table **S4**).

NRDEGs among the two sample groups (IH/Normal) in the GSE127487 dataset were analyzed utilizing the pheatmap package in R. These findings are illustrated in the differential ranking chart shown in Fig. (**3c**), which displays the ordered differences of the NRDEGs in the GSE127487 dataset. The sorting was based on the absolute log fold change value (|logFC|) of each NRDEG, which was obtained from the difference analysis between the IH group and the normal group by the limma package. Larger values of |logFC| indicated higher levels of differential expression of the gene.

### 3.4 GO and KEGG Enrichment Analyses

We further explored BP, CC, and MF of the 74 NRDEGs, along with their connection to biological pathways in IH through GO and KEGG enrichment analyses. Seventy-four NRDEGs were used to conduct comprehensive GO and KEGG enrichment analyses, the specific outcomes of which are detailed in Table **S5**. 

The analysis demonstrated significant enrichment of the 74 NRDEGs in various pathways. NRDEGs were notably enriched in pathways related to BP, including macroautophagy, I-kappa B kinase/NF-kappaB signaling, the stress-activated MAPK cascade, and cellular responses to hypoxia. Furthermore, enrichment in the CC pathway included focal adhesion, cell-substrate junctions, membrane microdomains, and the cytoplasmic side of the membrane. In terms of MF, NRDEGs exhibited enrichment related to ubiquitin protein ligase binding, cysteine-type endopeptidase activity involved in apoptosis-related signaling pathways, death receptor activity, and cysteine-type endopeptidase activity implicated in the apoptotic process.

Simultaneously, KEGG analysis also displayed enrichment in various biological pathways, including the pathogenic *Escherichia coli* infection pathway, IL-17 signaling pathway, necroptosis, MAPK signaling pathway, autophagy (animal), NF-κB signaling pathway, apoptosis, and toxoplasmosis (KEGG). Bubble plots [33] (Figs. **4a**–**b**) present the outcomes of the GO and KEGG enrichment analyses.

Network diagrams were constructed utilizing the data derived from the GO and KEGG enrichment analyses, demonstrating the relationships within the MF, BP, CC, and biological pathways (Figs. **4c**–**d**). The lines connecting the nodes signify the connections between molecules, with annotations providing details of the corresponding entries. Larger nodes denote entries linked to a higher number of molecules.

### 3.5 GSEA and GSVA

The influence of gene expression levels in the GSE127487 dataset was investigated using GSEA, focusing on the expression and biological mechanisms of all genes in GSE127487, the impacted cellular elements, and the correlations among molecular activities (Fig. **5a**). The summarized results are presented in Table **S6**. Notably, all the genes in the GSE127487 dataset showed significant enrichment in biologically relevant functions and signaling pathways, such as the FcεR1 pathway (Fig. **5b**), Notch pathway (Fig. **5c**), glycolysis pathway (Fig. **5d**), IL12 pathway (Fig. **5e**), and Wnt pathway (Fig. **5f**).

To compare the h.all.v7.4. symbols.gmt gene set across different groups (IH/Normal) within the GSE127487 dataset, all the genes present in the dataset were subjected to GSVA. The detailed findings are presented in Table **S7**. The GSVA outcomes revealed statistically significant differences in various pathways across different groups (IH/Normal) of the GSE127487 dataset, including fatty acid metabolism, adipogenesis, estrogen response (early/late), oxidative phosphorylation, and heme metabolism (*p* < 0.05). Based on the GSVA results, the two groups (IH/Normal) in the GSE127487 dataset were subjected to differential expression analysis, and the findings were presented visually through heatmaps (Fig. **5g**).

### 3.6 Development of the Diagnostic Model for IH

A single-factor logistic regression analysis using the 74 identified NRDEGs was conducted to assess their diagnostic significance in IH. Our findings revealed that 44 NRDEGs exhibited statistically significant results (*p* < 0.05, Table **S8**).

Subsequently, a LASSO regression analysis was performed to create an IH diagnostic model utilizing the 44 NRDEGs. A visual display of the results was created by drawing the LASSO regression model diagram (Supplementary Fig. **S1a**) and the LASSO variable trajectory diagram (Supplementary Fig. **S1b**). The analysis revealed that out of the initial 44 NRDEGs, eight genes were identified as model genes within the LASSO regression model: *CHMP4B, EPAS1, LIMCH1, PDIA6, TANC2, TMEM44, TP53I3,* and *UHRF1*.

Following this, an SVM model was developed utilizing the SVM algorithm and 44 NRDEGs. The model was evaluated based on gene count with the lowest error rate (Supplementary Fig. **S1c**) and the highest accuracy (Supplementary Fig. **S1d**). According to the findings, maximum accuracy in the SVM model was achieved when the gene count was 21. Subsequently, 21 NRDEGs were identified as: *SKP2, ADAR, PELI1, TNFRSF10A, RBCK1, HPRT1, TMEM44, CHMP4B, EPAS1, CASP3, SLC25A10, LIMCH1, MPRIP, TP53I3, HNRNPM, ZC3HAV1, FIP1L1, CASP2, NDUFA4, TANC2,* and *MYO1D*.

Finally, six NRDEGs (key genes) were identified for subsequent analysis by considering the intersection of the NRDEGs in both the LASSO regression model and the SVM model, resulting in the creation of a Venn diagram (Supplementary Fig. **S1e**). The six key genes identified were *CHMP4B, EPAS1, LIMCH1, TANC2, TMEM44,* and *TP53I3.* Subsequently, a LASSO regression analysis was performed to create an IH diagnostic model utilizing the 44 NRDEGs. A visual display of the results was created by drawing the LASSO regression model diagram (Supplementary Fig. **S1a**) and the LASSO variable trajectory diagram (Supplementary Fig. **S1b**). The analysis revealed that out of the initial 44 NRDEGs, eight genes were identified as model genes within the LASSO regression model: *CHMP4B, EPAS1, LIMCH1, PDIA6, TANC2, TMEM44, TP53I3*, and *UHRF1*. These genes were then used for subsequent analyses.

### 3.7 Validation of the Diagnostic Model for IH

To confirm the effectiveness of the IH diagnostic model, a nomogram derived from the model genes was created to illustrate the connections between the model genes in the GSE127487 dataset (Fig. **6a**). After excluding genes with no results, the expression of the model gene *TP53I3* was found to be more effective in the diagnostic model of IH.

A DCA [34] was utilized to investigate the clinical significance of the IH diagnostic model based on the model genes in the GSE127487 dataset (Fig. **6b**). It was found that the line of the model consistently exceeded both positive and negative values within a specified range. Additionally, the net income of the model was greater, suggesting that the model was more effective.

The ROC curve [35] was plotted between the linear predictors of the logistic regression model for the two groups (IH/Normal) in the GSE127487 dataset, with the results displayed in Fig. (**6c**). This figure illustrates that the logistic regression model of the GSE127487 dataset has a reliable diagnostic effect.

### 3.8 Analysis of Expression Differences of the Key Genes between the Various Groups in the GSE127487 Dataset

We used a group comparison violin plot (Fig. **7a**) to show the difference in the analysis results for the expression of the six key genes between the two groups (Normal/IH) in the GSE127487 dataset. Notably, the expression levels of four key genes (*CHMP4B, EPAS1, TANC2,* and *TMEM44*) differed significantly between the two groups (Normal/IH) in the GSE127487 dataset (*p* < 0.001). Additionally, the levels of the other two key genes, *LIMCH1* and *TP53I3,* between the two groups (Normal/IH), were also highly significant (*p* < 0.05).

Utilizing the full expression matrix of the six identified key genes from the GSE127487 dataset, a correlation analysis was performed, and a correlation heat map was produced (Fig. **7b**). The data illustrated a positive association between the key genes *TANC2* and *CHMP4B,* as well as *TMEM44* and *EPAS1*. Conversely, a negative association was noted between the key genes *EPAS1* and *CHMP4B,* as well as *TANC2* and *TP53I3*.

Finally, the results of the GSE127487 dataset were displayed using ROC curves for the six key genes (Fig. **7c**–**h**). Notably, the expression variances of the six key genes in the GSE127487 dataset demonstrated notable precision (AUC > 0.9) across the two groups, as demonstrated by the ROC curve.

### 3.9 Networks Illustrating mRNA-miRNA as well as mRNA-TF Interactions

miRNAs that interacted with the six key genes were predicted by analyzing the mRNA-miRNA data utilizing the ENCORI database. Subsequently, the database results and 38 DEGs (miRNAs) from the GSE100682 dataset were combined to identify the shared miRNAs. A Venn diagram (Fig. **8a**) revealed six common miRNAs (hsa-miR-515-5p, hsa-miR-520d-5p, hsa-miR-525-3p, hsa-miR-518b, hsa-miR-518a-3p, and hsa-miR-520c-3p). The Cytoscape program was utilized to visually display the mRNA-miRNA interaction network (Fig. **8b**), which illustrated the involvement of four key genes (*LIMCH1, TANC2, EPAS1,* and *CHMP4B*) and six miRNAs. The molecular composition constituted eight pairs of mRNA-RBP interactions (Table **S9**).

Finally, the CHIPBase database (v 3.0) was utilized to search for the TFs binding to the six key genes. Subsequently, the network of mRNA-TF interactions was created and depicted using Cytoscape (Fig. **8c**), which culminated in the acquisition of interaction data for five key genes (*CHMP4B, EPAS1, LIMCH1, TANC2,* and *TP53I3*) and 47 TFs, totaling 59 pairs [36]. Table **S10** presents the relationship between the mRNA and TF levels.

### 3.10 Immune Infiltration Analysis (ssGSEA and MCPCounter)

The association between the expression profiles of the 28 types of immune cells across the two groups (Normal/IH) in the GSE127487 dataset was determined *via* ssGSEA. Group comparison box plots were utilized to present the immune infiltration analysis results (Fig. **9a**), showing the abundance of 28 types of immune cells across the two groups. The findings highlighted significant (*p* < 0.05) variances in the expression levels of five types of immune cells across the two groups (Normal/IH) in the GSE127487 dataset: gamma-delta T cells, immature DCs, NK T cells, activated CD8 T cells, and neutrophils.

The levels of immune infiltration for the five statistically significant (*p* < 0.05) genes among six key genes were visually represented using a correlation heatmap (Fig. **9b**). The analysis revealed a positive association between *TMEM44* and NK T cells, as well as *EPAS1* and gamma-delta T cells. There was a negative association between *TP53I3* and immature DCs, as well as *LIMCH1* and gamma-delta T cells.

Further analysis was carried out to examine the association between six key genes and immune cell infiltration abundance across the two groups (Normal/IH) in the GSE127487 dataset, as shown in Fig. (**9c**). Notably, these key genes were found to be related to eight types of immune cells, notably, B lineage, NK cells, fibroblasts, myeloid DCs, monocytic lineage, neutrophils, endothelial cells, and T cells. Furthermore, key genes *EPAS1* and *TMEM44* were positively related to endothelial cells, whereas *TANC2* and *EPAS1* were negatively related to these immune cells.

## 4 DISCUSSION

Necroptosis is a form of programmed cell death that operates independently of apoptosis. This process is primarily driven by the activation of mixed-lineage kinase domain-like pseudokinase, receptor-interacting protein kinase 1, and receptor-interacting protein kinase 3 [37]. Necroptosis is found to participate in the pathogenesis of various diseases, including cancer [38]. Based on bioinformatic analyses, a recent study identified two clusters associated with necroptosis and a prognostic signature consisting of 10 genes, enabling the optimization of risk stratification and treatment of hepatocellular carcinoma [39].

IH is the most prevalent benign neoplasm in children, and its detrimental effects on the patient's appearance are significant, as tissue and organ malformations and dysfunction can reduce the quality of life of patients and can even lead to psychological disorders. Tumor pathogenesis and progression are both closely associated with necroptosis. However, the role of NRGs in IH remains unclear, as limited research has been conducted in this area.

We conducted statistical analyses and generated graphs by utilizing the R software to analyze the IH datasets in the GEO database. The results revealed that several NRGs showed significant differential expression levels between the samples in the IH and normal groups. In addition, in-depth functional and pathway enrichment analyses demonstrated the biological processes and pathways in which NRGs might be involved in IH pathogenesis. We identified 74 NRDEGs in the GSE127487 and GSE100682 datasets. Among them, 44 NRDEGs were identified as statistically significant for IH by logistic regression analysis, providing the basis for subsequent diagnostic model construction. This suggests that necroptosis is critically involved in the IH pathogenesis. We constructed a LASSO diagnostic model utilizing 44 NRDEGs and screened eight model genes. Similarly, SVM analysis identified 21 NRDEGs, from which six key genes were identified (*CHMP4B, EPAS1, LIMCH1, TANC2, TMEM44,* and *TP53I3*). The identification of these key genes not only revealed the potential role of necroptosis in IH but also provided new molecular markers for clinical diagnosis.

Charged multivesicular body protein 4 B (*CHMP4B*) serves as a vital component of the endosomal sorting complex required for transport (ESCRT)-III and is implicated in cytokinetic membrane abscission as well as the later stages of mitotic cell division. Endothelial PAS domain-containing protein 1 (EPAS1) functions as an oxygen-sensitive element of hypoxia-inducible factor 1 (HIF-1). Herein, we discovered a strong association between the loss of C expression and advanced tumor stage, as well as an overexpression of vascular endothelial growth factor and *in vitro* angiogenesis [40]. The results of regulatory network analysis revealed two B-associated miRNAs and one C-associated miRNA. Moreover, TF analysis revealed 13 B-associated TFs and four C-associated TFs. Overall, this data suggests that key genes may include those implicated in cell proliferation and apoptosis, and that aberrant expression levels of these genes may contribute to the formation of the hemangioma cell microenvironment, which subsequently influences the proliferation, migration, and prognosis of tumor cells.

The GO and KEGG enrichment analyses revealed that the NRDEGs were predominantly associated with the IκB kinase/NF-κB signaling pathway. NF-κB is recognized for its role in regulating cellular death and survival choices. Activation of NF-κB leads to its translocation into the cell nucleus, triggering gene transcription for various cellular processes, such as cell death, survival, and growth [41]. In contrast, NRDEGs influence IH development by participating in the NF-kappaB signaling pathway. Therefore, necroptosis is crucial for IH. Necroptosis regulates pro-inflammatory cytokine production, leukocyte recruitment, and cell survival by affecting the NF-κB pathway. Additionally, the activation of the NF-κB pathway is closely linked to the secretion of inflammatory factors and angiogenesis. Moreover, enhanced glycolytic metabolism can promote the abnormal proliferation and angiogenesis of vascular endothelial cells, which plays an important role in the development of IH [42]. These factors are important in the inflammatory response and greatly influence endothelial cell production and apoptosis.

In the GSEA, the glycolysis pathway was the most significantly enriched signaling pathway. Endothelial cells (ECs) play a crucial role in angiogenesis and vasculogenesis, which are essential processes that drive the formation of new blood vessels during IH. These intricate processes require substantial energy to support the proliferation and migration of ECs. Recent studies have highlighted the significant contribution of endothelial cell metabolic activity, particularly glycolytic metabolism, in promoting angiogenesis [43]. In a study by Yang *et al.*, PKM2, an essential glycolytic enzyme, was shown to stimulate angiogenesis in hemangioma ECs (HemECs). Conversely, PKM2 inhibition has been shown to inhibit IH advancement in mouse models [44]. Likewise, an additional study revealed that by selectively targeting PFKFB3, glycolysis-induced apoptosis in HemECs is impeded, thereby effectively inhibiting angiogenesis [45]. Li *et al.* revealed that glycolysis was inhibited when TGFBI was silenced in HemECs, demonstrating that TGFBI promotes angiogenesis and progression in HemECs by regulating glycolysis [46]. In summary, NRGs can influence the migration and proliferation of IH endothelial cells by modulating glycolytic pathways. Considering the above criteria, directing therapeutic efforts toward glycolysis is possible to inhibit the progression of IH and angiogenesis.

In the interaction network analysis, an interactive relationship among six key genes, miRNAs, and TFs was found. By analyzing the network interactions of mRNA-miRNA and mRNA-TF, we were able to reveal the complex network of gene expression regulation and identify potential regulatory pathways. Therefore, this study presents novel insights into the molecular mechanisms underlying IH and highlights potential targets for the development of future targeted therapeutic regimens.

Tumor cell proliferation occurs within a complex microenvironment comprising stromal, immune, and cancer cells. Tumor progression is intricately intertwined with the tumor microenvironment and is characterized by dynamic and reciprocal interactions. Tumor cells can modulate their microenvironment by releasing signaling molecules that stimulate tumor angiogenesis and foster immune tolerance. Simultaneously, the immune cells within the microenvironment can influence the growth and progression of tumor cells. Furthermore, the presence of infiltrating immune cells is a predictor of neoadjuvant chemotherapy and immune checkpoint inhibitors (ICIs) treatment outcomes [47]; therefore, screening for infiltrating immune cells is not only useful for combined ICI therapy but also has potential predictive value for the same. In this study, immune infiltration was analyzed because enrichment analysis revealed genes enriched in signaling pathways associated with leukocyte migration and immune response regulation. The immune infiltration analysis revealed that certain key genes were positively linked to the abundance of immune cells. The correlation dot plots of immune cell types and key genes supported this finding. We discovered a positive correlation between TMEM44 and NK T cells, EPAS1, gamma-delta T cells, TP53I3, and immature DCs, as well as LIMCH1 and gamma-delta T cells. Conversely, TP53I3 and immature DCs, as well as LIMCH1 and gamma-delta T cells, exhibited certain negative correlations. This reveals the immunopathological mechanism of IH from the perspective of the immune microenvironment and provides clues for immune-related therapy.

The molecular mechanisms of propranolol, the drug of choice for the non-surgical treatment of IH, have been confirmed by several studies [48], primarily β-adrenergic receptor (β-AR) blockade, which affects the downstream cAMP/PKA signalling pathway [49], inhibition of the expression of pro-angiogenic molecules, such as vascular endothelial growth factor (VEGF) [50], and regulation of cellular signaling pathways by interfering with MAPK/ERK [51], PI3K/AKT [52], *etc*., thereby affecting proliferation, apoptosis, and angiogenesis. In addition, propranolol may exert anti-tumour effects by regulating inflammatory responses and cellular metabolic pathways [53].

Compared with the six key gene-related signalling pathways screened in this study, we found that pathways, such as NF-κB, MAPK, Notch, Wnt, and glycolysis, overlapped with the known pathways of propranolol [54], especially in the regulation of neovascularisation, inflammatory response, and apoptosis [55, 56]. Notably, the NF-κB and MAPK signalling axes were both downstream of propranolol's action [57] and pathways enriched for key genes in this study, suggesting that these genes may mediate some of the biological effects of propranolol in the treatment of IH. In addition, the enrichment of glycolytic and metabolic pathways reveals the role of metabolic reprogramming in tumour cells in IH progression and treatment [58]. Therefore, the molecular markers we identified not only contribute to the early and precise diagnosis of IH but also provide new potential targets for patients who are unresponsive or resistant to conventional therapy, thereby promoting the development of molecular typing and individualized treatment of IH. With the deeper understanding of the molecular mechanisms of IH, drug selection and combination therapy based on key pathways are expected to be realised in the future, thus improving clinical management.

This study is mainly based on the analysis of gene expression data, and the biological functions of the six key genes in normal vascular endothelial cells have not yet been experimentally verified. Therefore, the specific roles of these genes in normal vascular endothelial cells and their safety as potential therapeutic targets still need to be further elucidated in subsequent cell and animal experiments. In addition, there is no direct evidence on the expression of these genes in healed wound tissues and their effects on the healing process, and their roles in tissue repair and vascular regeneration can be further explored in combination with relevant models in the future [59, 60].

Although this study has made significant progress in identifying and validating differentially expressed genes associated with necrotic apoptosis in infantile hemangiomas, several limitations remain. The samples analysed in this study were mainly derived from hemangioma tissues obtained by surgical resection or ablative treatment. Surgical or ablative interventions remain the primary treatment for focal, easily surgical or laser-treatable lesions. However, genetic analysis helps to reveal the molecular mechanisms, especially in complex cases that cannot be surgically resected, recur, or are refractory to conventional treatment, and provides an important reference for individualised diagnosis and the development of treatment protocols. Therefore, our findings have implications for clinical translational applications in complex or refractory cases, but their clinical applicability should be interpreted in the context of the sample source. Future studies should incorporate more independent datasets, including data from non-surgically treated patients, to further validate the validity of the model and expand its clinical applicability.

In this study, two datasets, GSE127487 and GSE100682, from the GEO database were used for analysis of the data, construction of diagnostic models, and screening of key genes. Although we demonstrated the accuracy and robustness of the model through cross-validation, given the limited number of existing publicly available IH high-throughput datasets with small sample sizes and the lack of external independent validation sets, this may have some impact on the generalisability and stability of the study results. It should be noted that all the results were derived from bioinformatics predictions, which have not been functionally validated by qPCR, Western blot, and other experiments or clinical samples to date due to time and resource constraints. We plan to conduct expression validation and mechanistic exploration of key genes in combination with tissue samples in subsequent studies to further support the conclusions of this study. In addition, the immune infiltration correlation analysis only revealed a correlation between the key genes and certain immune cells, and could not yet prove a direct causal role. This will be followed up with *in vitro* and *ex vivo* functional experiments, as well as a literature review, to strengthen the biological plausibility.

## CONCLUSION

In this study, we employed bioinformatics to comprehensively investigate the pathogenesis of IH, construct a diagnostic model, and screen for six key NRDEGs (*CHMP4B, EPAS1, LIMCH1, TANC2, TMEM44*, and *TP53I3*). The development, progression, and metastasis of IH were all found to be linked to the expression of these genes. It is worth noting that the primary mechanism of action of propranolol, as a first-line therapeutic agent for IH, involves β-adrenergic receptor blockade, inhibition of angiogenesis, and induction of vasoconstriction, which does not completely overlap with the programmed necrosis and related immune signaling pathways revealed in this study. In conclusion, the molecular markers identified enable early and precise diagnosis of IH and present promising targets for patients unresponsive to conventional therapies, paving the way for improved molecular classification and personalized treatment approaches. With a deeper understanding of the molecular mechanisms of IH, future drug selection and combination therapy based on key pathways are expected to enhance clinical management.

## Figures and Tables

**Fig. (1):**
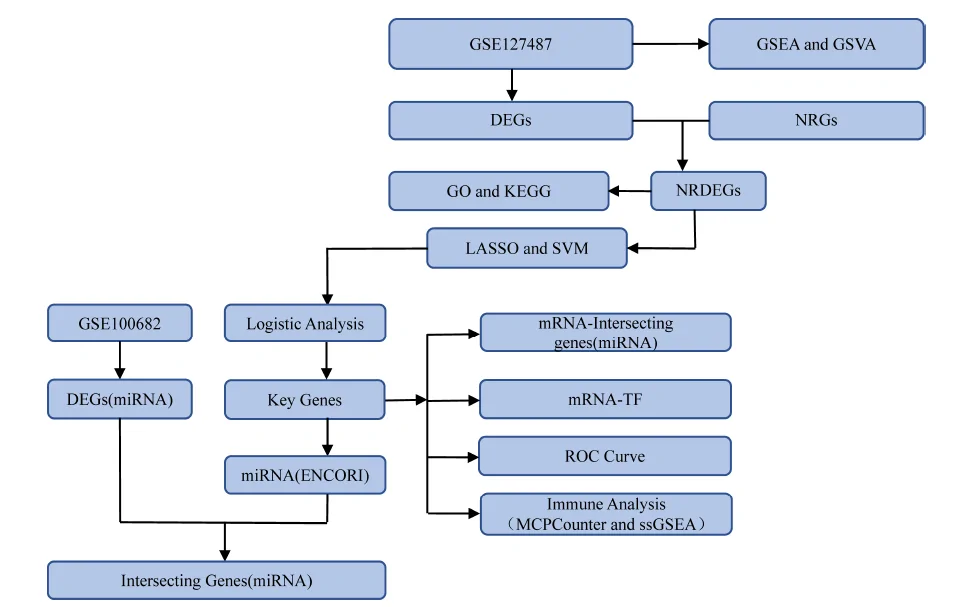
Technology roadmap. **Abbreviations:** PCA: Principal Component Analysis; GSEA: Gene Set Enrichment Analysis; GSVA: Gene Set Variation Analysis; NRGs: Necroptosis-Related Genes; DEGs: Differentially Expressed Genes; NRDEGs: Necroptosis-Related Differentially Expressed Genes; SVM: Support Vector Machine; GO: Gene Ontology; KEGG: Kyoto Encyclopedia of Genes and Genomes; LASSO: Least Absolute Shrinkage and Selection Operator; ROC: Receiver Operating Characteristic; TF: Transcription Factor.

**Fig. (2):**
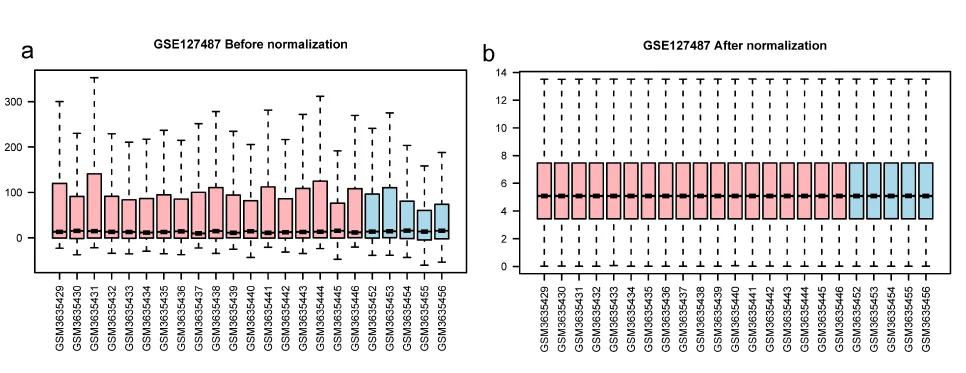
Dataset De-Batch processing. (**a**) Boxplot of gene expression distribution among the GSE127487 samples before correction. (**b**) Box plot of the corrected gene expression distribution in the GSE127487 samples. Pink and blue colors denote the IH and normal groups in the dataset GSE127487, respectively.

**Fig. (3):**
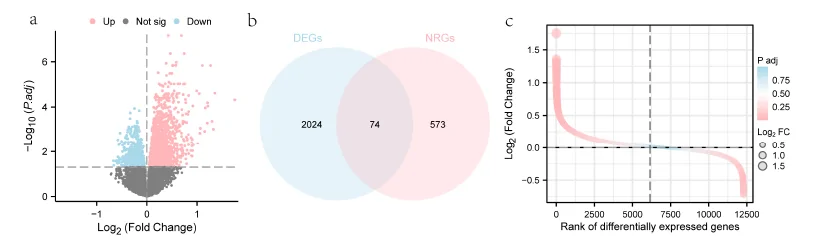
Differential gene expression analysis. (**a**) Gene expression analysis between the IH and normal groups in the GSE127487 dataset, as depicted in a volcano plot. (**b**) The DEGs and NRGs in the GSE127487 dataset are displayed in a Venn diagram. (**c**) Differential ranking diagram of the NRDEGs in dataset GSE127487. **Abbreviations:** DEGs, Differentially Expressed Genes; NRGs, Necrosis-related Genes; NRDEGs, Necrosis-Related Differentially Expressed Genes.

**Fig. (4):**
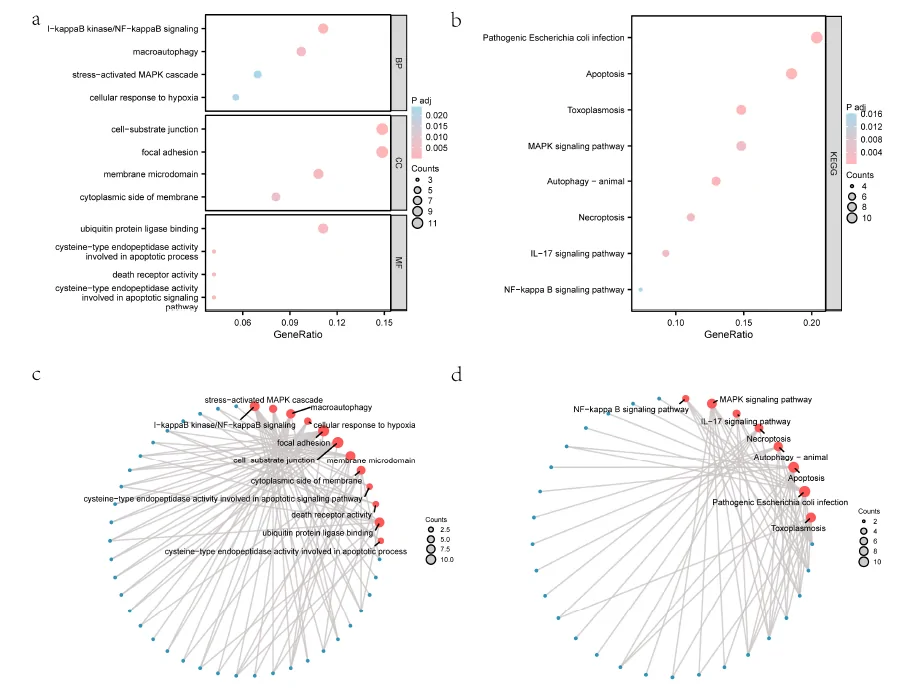
GO and KEGG enrichment analyses for NRDEGs. (**a–b**) Bubble chart displays the outcomes of GO and KEGG enrichment analyses for the NRDEGs, including BP, CC, and MF pathways. The abscissa is GO terms and KEGG terms. (**c–d**) Network diagram showing the GO and KEGG enrichment analyses findings of the NRDEGs: BP, CC, MF (**c**), and KEGG (**d**). The entries and molecules are, respectively, denoted by the red and blue nodes, while the relationship between them is denoted by the connecting lines. **Abbreviations:** NRDEGs, Necroptosis-Related Differentially Expressed Genes; GO, Gene Ontology; KEGG, Kyoto Encyclopedia of Genes and Genomes; MF, Molecular Function; CC, Cellular Component; BP, Biological Process. An adj. *p* < 0.05 and FDR value (q-value) < 0.05 were chosen as the screening criteria for GO and KEGG analyses.

**Fig. (5):**
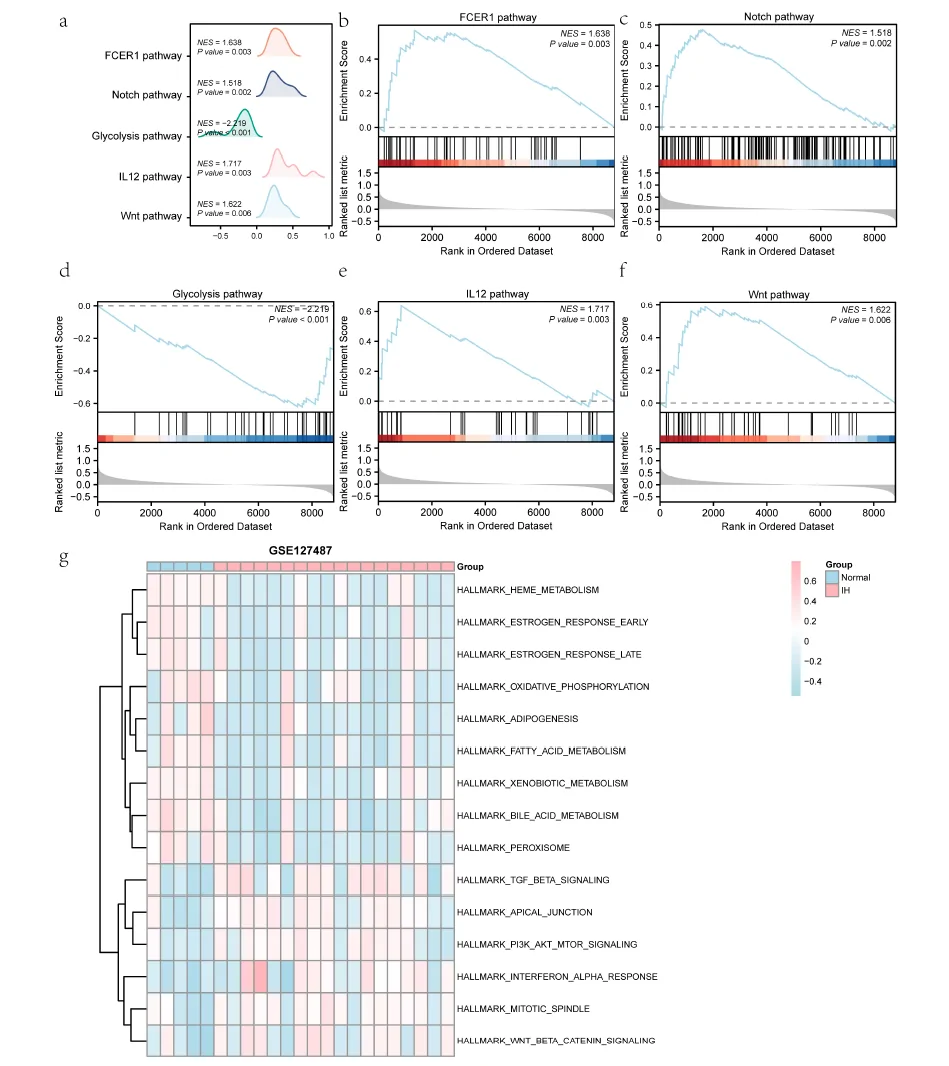
GSEA and GSVA for dataset GSE127487. (**a**) Mountain plot displays five biological functions of NRDEGs in the GSE127487 dataset based on GSEA. (**b-f**) GSEA showed that the NRDEGs were significantly enriched in the FcεR1 (**b**), Notch (**c**), glycolysis (**d**), IL12 pathway (**e**), and Wnt pathways (**f-g**) Based on the GSVA results, the two groups (IH/Normal) in the GSE127487 dataset were subjected to differential expression analysis. NRDEGs, necroptosis-related differentially expressed genes. GSEA, gene set enrichment analysis. GSVA, gene set variation analysis. An adj. *p* < 0.05 and FDR value (q-value) < 0.25 were used as the screening criteria for GSEA, while *p* < 0.05 and an FDR value (q-value) < 0.25 were used for GSVA. Red and blue, respectively, indicate the IH and normal groups.

**Fig. (6):**
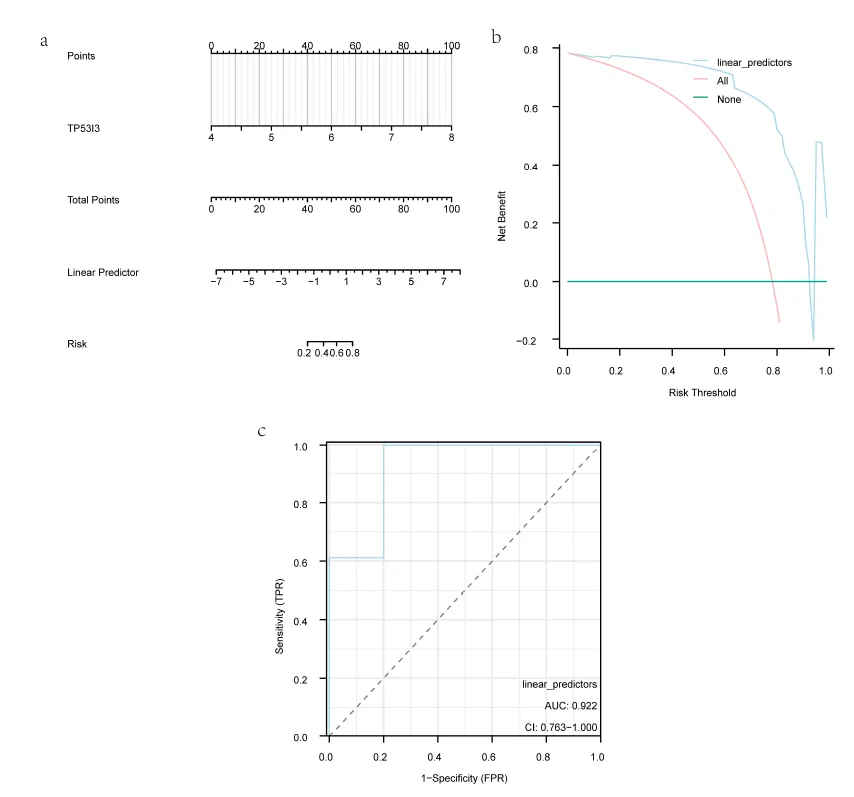
Diagnostic and validation analysis of IH. (**a**). A nomogram of model genes in the IH diagnostic model in the GSE127487 dataset. (**b**). A DCA diagram of the IH diagnostic model was created utilizing the model genes in the GSE127487 dataset. (**c**). ROC analysis of the linear predictors of the logistic regression model in the GSE127487 dataset. The ordinate of the calibration curve is the net income, and the abscissa denotes the probability threshold or threshold probability. AUC > 0.9 indicated a high accuracy. **Abbreviations:** AUC, Area Under the Curve. IH: Infantile Hemangioma. DCA: Decision Curve Analysis.

**Fig. (7):**
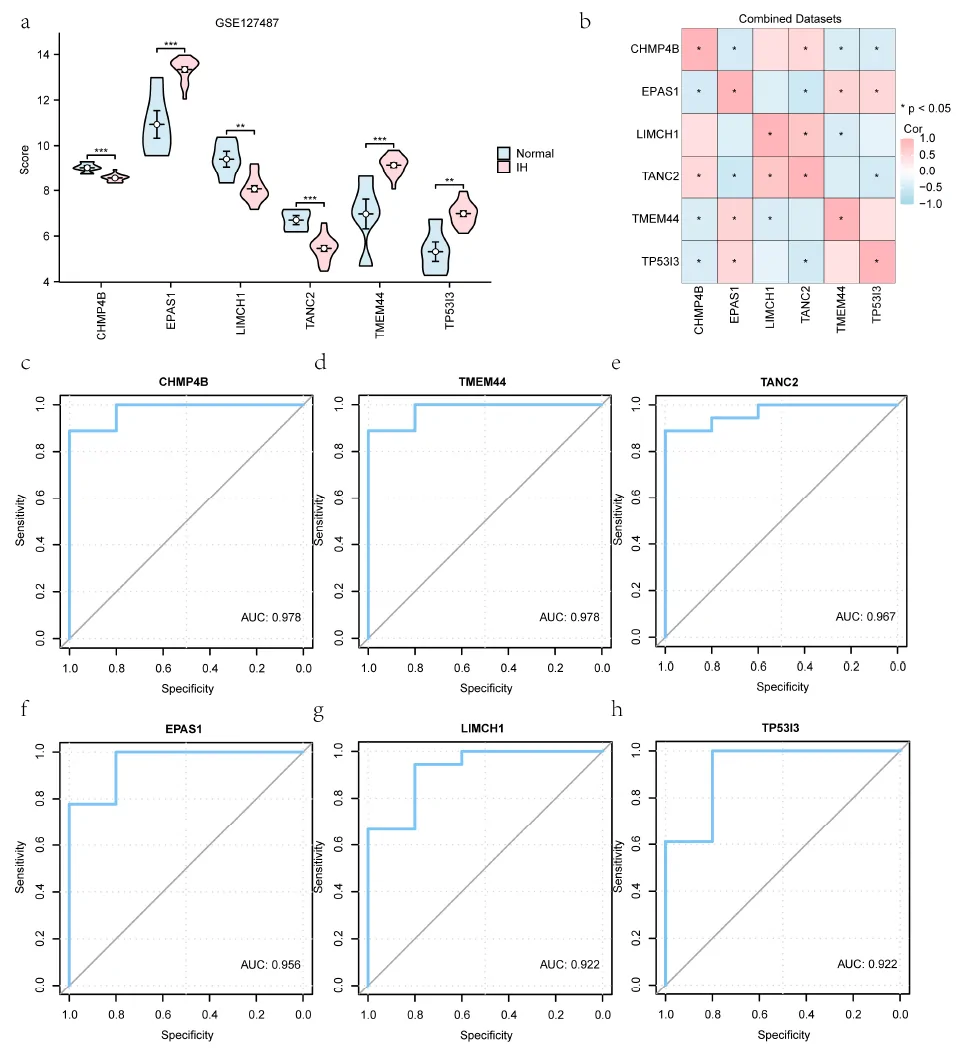
Analysis of expression differences in the key genes between the two groups in the GSE127487 dataset. (**a**). Group comparison chart of the key genes between the normal and IH groups in the GSE127487 dataset. (**b**). The key genes in the GSE127487 sample are presented in a correlation heatmap. ROC curve analysis of c–h. Key genes *CHMP4B* (**c**), *TMEM44* (**d**), *TANC2* (**e**), *EPAS1* (**f**), *LIMCH1* (**g**), and *TP53I3* (**h**) in the GSE127487 dataset. The symbol *** denotes a *p* < 0.001, which is highly significant; ** denotes *p* < 0.01, which indicates a high degree of significance; * denotes a *p* < 0.05, which demonstrates certain statistical significance. The diagnostic potential increases as the AUC of the ROC approaches a value of 1. The accuracy is higher when the AUC is > 0.9. AUC: Area Under Curve.

**Fig. (8):**
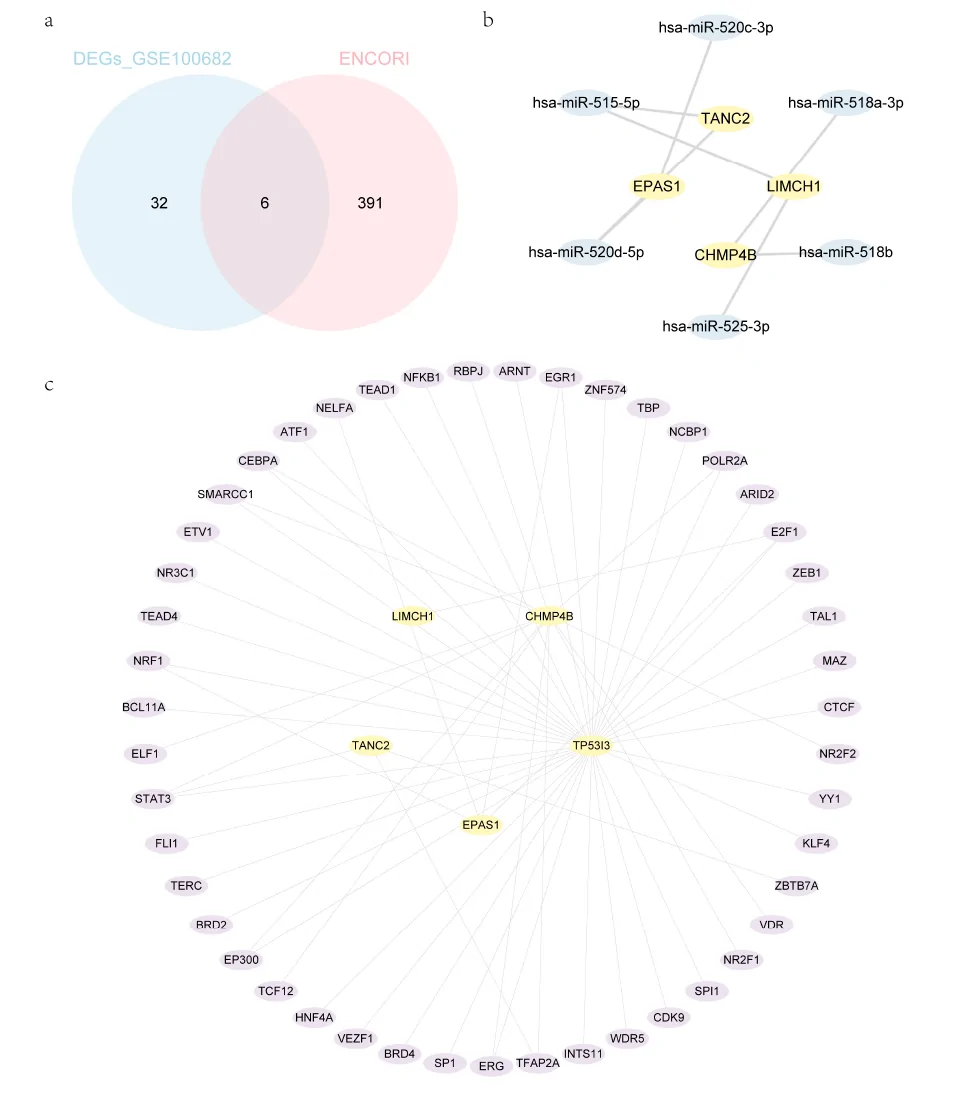
mRNA-miRNA and mRNA-TF interaction network. (**a**) Venn diagram representing the miRNA intersection of DEGs from GSE100682 and the ENCORI database. (**b**) Key gene-miRNA interaction networks. (**c**) Key gene-transcription factor interaction network. The mRNA, miRNA, and TFs are, respectively, denoted by a yellow oval, a blue ellipse, and a purple ellipse.

**Fig. (9):**
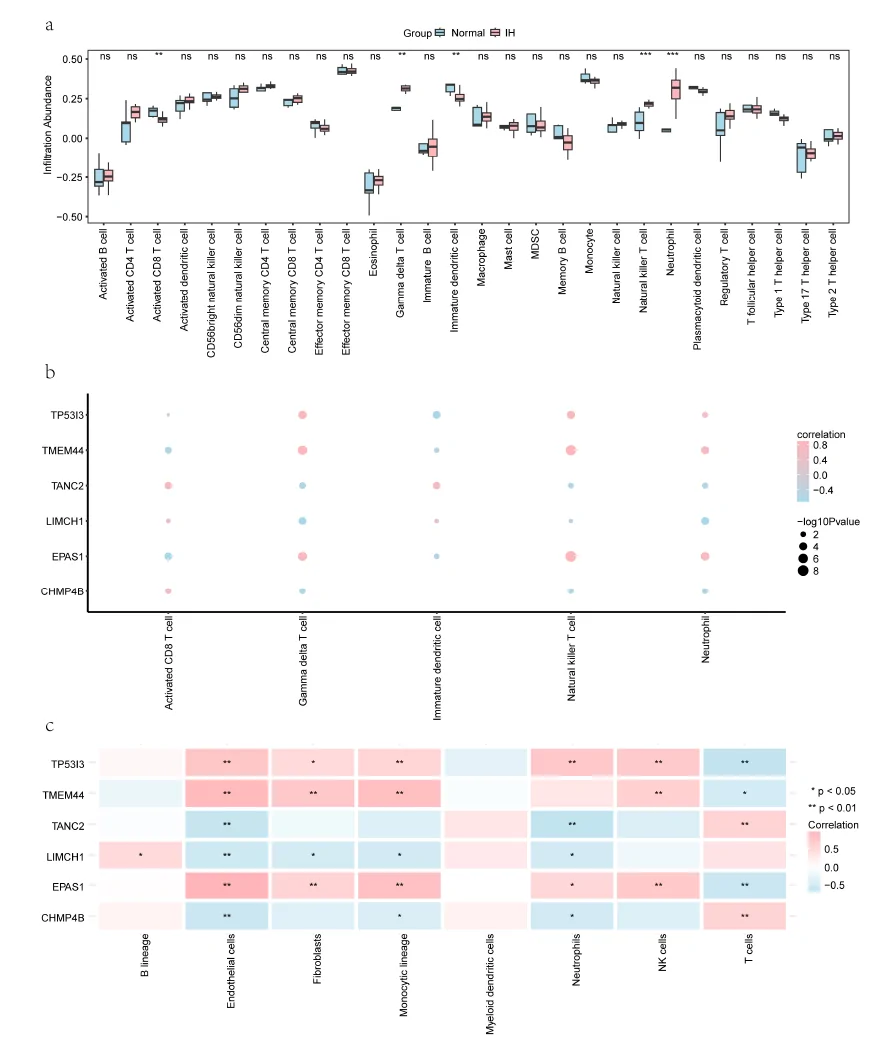
Immune infiltration analysis (ssGSEA and MCPCounter). (**a**) Group comparison chart between the two groups (Normal/IH) of 28 types of immune cells in the GSE127487 dataset under the ssGSEA algorithm. (**b**) Heat map showing the correlation between key genes and immune cell infiltration levels, as calculated using the ssGSEA algorithm. (**c**) The correlation analysis results between the key genes and immune cell infiltration levels, as calculated under the MCPCounter algorithm, are displayed in a heatmap. In the correlation heat map, the red circle illustrates a positive correlation between the gene and the infiltration abundance of immune cells, whereas a negative correlation is indicated by a blue circle, with the strength of the correlation increasing as the circle becomes larger. ns = *p* > 0.05, indicating no statistical significance; * denotes *p* < 0.05, indicating statistical significance; ** denotes *p* < 0.01, indicating a high degree of statistical significance; *** denotes *p* < 0.001, indicating extremely statistically significant significance.

## Data Availability

The data presented in this study are openly available in the GEO database [https://www.ncbi.nlm.nih.gov/geo/query/acc.cgi?acc=GSE127487, https://www.ncbi.nlm.nih.gov/geo/query/acc.cgi?acc=GSE100682, (accessed on 30^th^, October 2023)].

## LIST OF ABBREVIATIONS

IH: Infantile Hemangioma
NRDEGs: Necroptosis-Related Differentially Expressed Genes
NRGs: Necroptosis-Related Genes
SVM: Support Vector Machine
LASSO: Least Absolute Shrinkage And Selection Operator
ROC: Receiver Operating Characteristic
DAMPs: Damage-Associated Molecular Patterns
GEO: Gene Expression Omnibus
ssGSEA: Single-Sample Gene Set Enrichment Analysis
DEGs: Differentially Expressed Genes
GO: Gene Ontology
BP: Biological Process
CC: Cell Component
MF: Molecular Function
KEGG: Kyoto Encyclopedia Of Genes And Genomes
GSEA: Gene Set Enrichment Analysis
GSVA: Gene Set Variation Analysis
MSigDB: Molecular Signatures Database
DCA: Decision Curve Analysis
AUC: Area Under The Curve
ChIP: Chromatin Immunoprecipitation
TFs: Transcription Factors
DCs: Dendritic Cells
NK: Natural Killer
PCA: Principal Component Analysis
CHMP4B: Charged Multivesicular Body Protein 4 B
ESCRT: Endosomal Sorting Complex Required For Transport
HIF-1α: Hypoxia-Inducible Factor 1-Alpha
EPAS1: Endothelial PAS Domain-Containing Protein 1
ECs: Endothelial Cells
HemECs: Hemangioma ECs
ICIs: Immune Checkpoint Inhibitors
β-AR: β-Adrenergic Receptor
VEGF: Vascular Endothelial Growth Factor
