# Supplementary material for: Analysis of Necroptosis-Related Genes and Immune Cell Infiltration in Infantile Hemangioma
Source: Endocr Metab Immune Disord Drug Targets. 2025 Sep 16;26:E18715303388166. doi: 10.2174/0118715303388166250901114424 (PMC13635964; doi:10.2174/0118715303388166250901114424)
Supplement: Supplementary file 1 [file EMIDDT-26-E18715303388166_SD1.pdf]

Supplementary Material

Analysis of Necroptosis-Related Genes and Immune Cell Infiltration in Infantile Hemangioma

Yunpeng Xu[1], Jiajie Chen[1], Zhihao Huang[1], Rui Zhao[1], Can Jin[1], Zichen Li[1] and Shengxiu Liu[1,*]

[1]Department of Dermatovenerology, The First Affiliated Hospital of Anhui Medical University, Hefei, Anhui, China

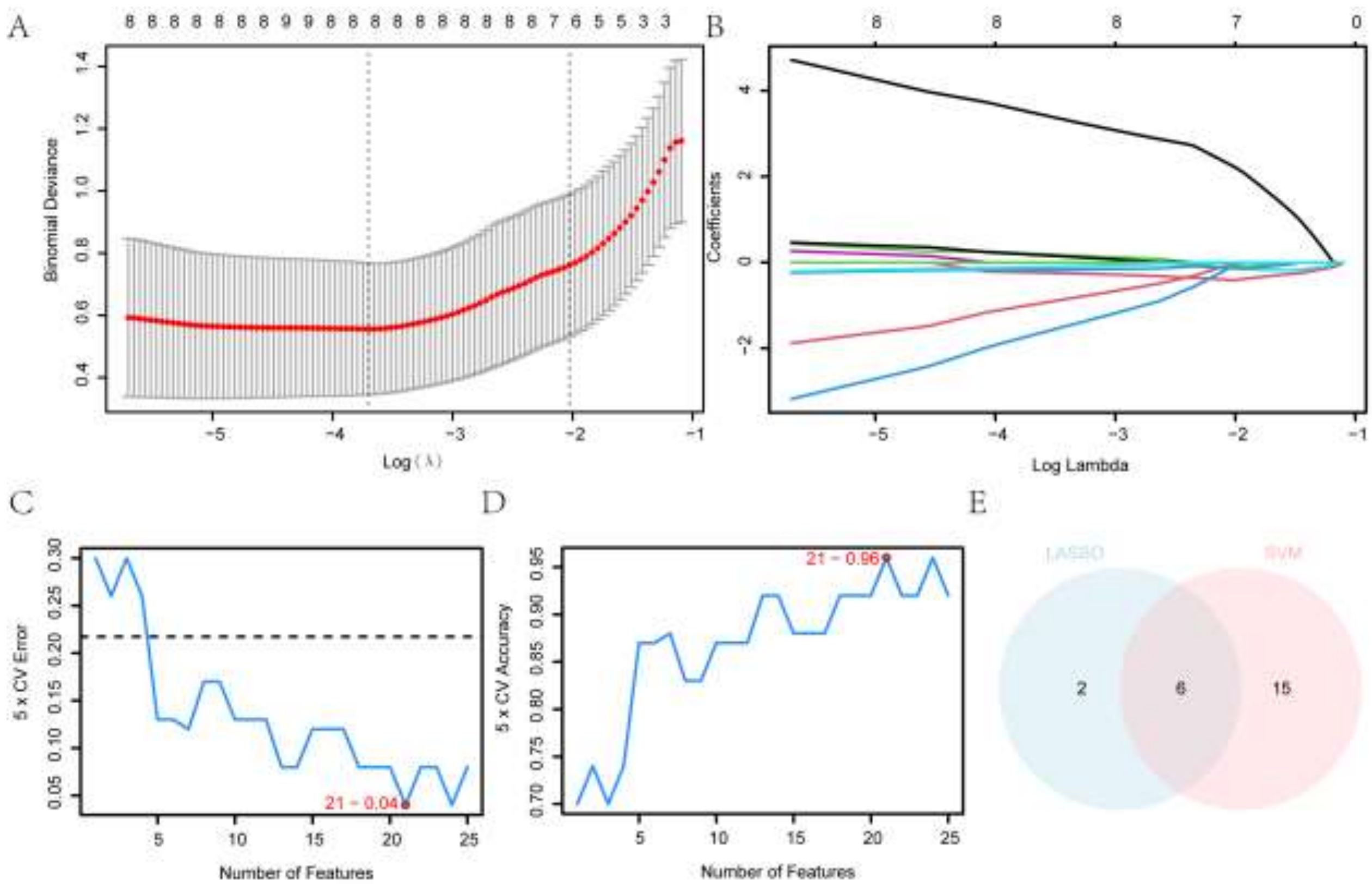

Supplementary Fig. (S1). Construction of diagnostic model for infantile hemangioma.
